# Palatine Tonsillar Metastasis of Small-Cell Neuroendocrine Carcinoma from the Lung Detected by FDG-PET/CT After Tonsillectomy: A Case Report

**DOI:** 10.5812/iranjradiol.9281

**Published:** 2013-08-30

**Authors:** Xiao-Hong Chen, Yang-Yang Bao, Shui-Hong Zhou, Qin-Ying Wang, Kui Zhao

**Affiliations:** 1 Department of Otolaryngology, the First Affiliated Hospital, College of Medicine, Zhejiang University, Hangzhou, Zhejiang, China; 2 Department of Otolaryngology, the Second Hospital of Jiaxing City 314000, Zhejiang, China; 3 Center of PET, the First Affiliated Hospital, College of Medicine, Zhejiang University, Hangzhou, Zhejiang, China

**Keywords:** Palatine Tonsil, Carcinoma, Neuroendocrine, Lung

## Abstract

Metastasis from a malignant tumor to the palatine tonsils is rare, accounting for only 0.8% of all tonsillar tumors, with only 100 cases reported in the English-language literature. Various malignant lung carcinomas may metastasize to the tonsils. A few cases of tonsillar metastasis from neuroendocrine lung carcinoma have been reported. A 67-year-old female underwent a right tonsillectomy because of a sore throat and an enlarged right tonsil. The postoperative pathology showed right tonsillar small cell neuroendocrine carcinoma (SCNC). Fluorodeoxyglucose (FDG) positron emission tomography (PET)/computed tomography (CT) demonstrated metabolic activity in the lower lobe of the right lung. In addition, hypermetabolic foci were noted in the lymph nodes of the right neck and mediastinum. A needle biopsy of the pulmonary mass showed SCNC. The patient received chemotherapy and died of multiple distant metastases after 6 months. This is the first report using PET/CT to evaluate tonsillar metastasis from lung SCNC.

## 1. Introduction

Metastasis from a malignant tumor to the palatine tonsils is rare, accounting for only 0.8% of all tonsillar tumors (1), with only 100 cases reported in the English-language literature (2, 3). The most common primary sites are the breast (1), stomach (2), intestinal tract (3), cutaneous melanoma (4) and the kidney (5). Various malignant lung carcinomas may metastasize to the tonsils (6, 7) and a few cases of tonsillar metastasis from neuroendocrine lung carcinoma have been reported (6, 7). Here, we present an additional case of metastasis to the palatine tonsils from a small-cell neuroendocrine carcinoma (SCNC) in the lung. To our knowledge, our patient is the first report of the use of positron emission tomography/computed tomography (PET/CT) in evaluating tonsillar metastasis from lung SCNC after tonsillectomy.

## 2. Case Presentation

**Figure 1.**
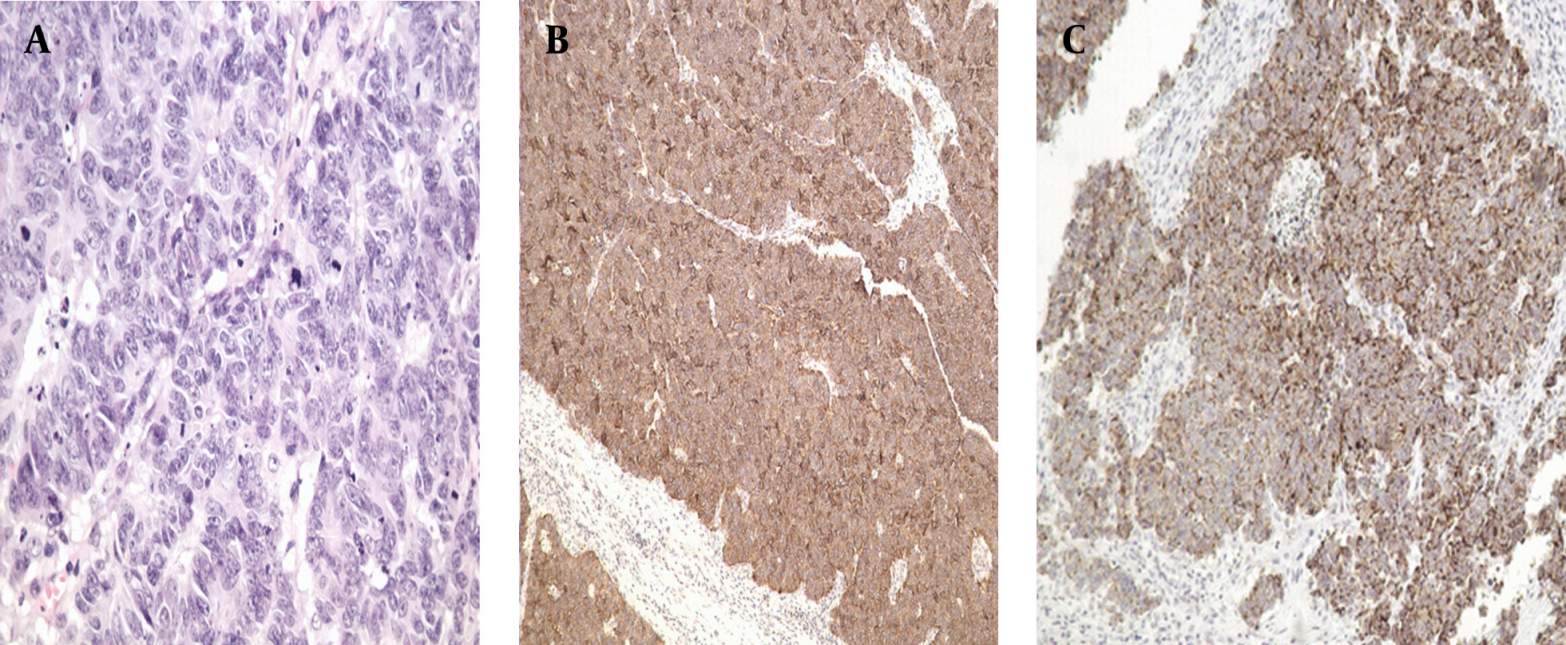
Pathology shows the lesion with small tumor cells arranged in irregular nests with infiltrative growth and increased mitotic activity (A: H&E×400). Immunohistochemically, the tumor was positive for CD56 (B: EliVision™× 100), and CHGA (C: EliVision™× 100).

**Figure 2.**
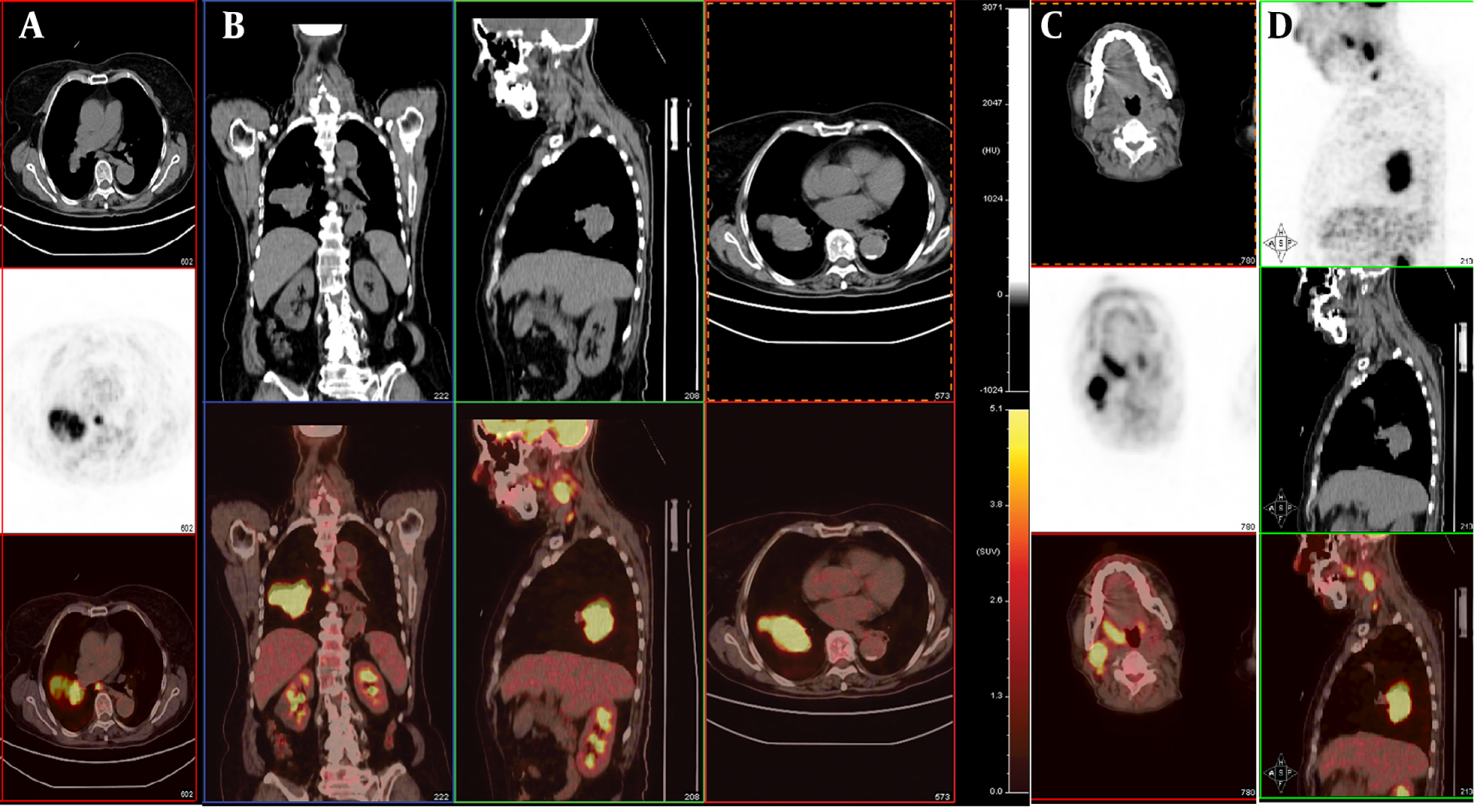
A and B, PET/CT images reveal multiple regions of increased FDG activity in the right lung (SUVmax=11.6) and mediastinal lymph nodes (SUVmax=7.9). C, The images show increased FDG uptake in the right oropharynx (SUVmax=7.3) and multiple intensely 18F-FDG-avid right submaxillary lymph nodes (SUVmax=13.6). D, The image shows increased FDG uptake in the oropharynx

Written informed consent was obtained from the patient’s son for publication of this report and any accompanying images. Institutional ethical approval was obtained. In September 2011, a 67-year-old female presented with a sore throat and an enlarged right tonsil of half a month duration, resulting in swallowing difficulties. She had a fever (body temperature up to 38.4°C). There was no peritonsillar abscess on puncture. The fever disappeared after antibiotic treatment for 5 days. However, the pharyngalgia did not improve and the enlarged right tonsil changed markedly. Her medical history included a cholecystectomy for cholelithiasis 10 years earlier and a double knee arthroplasty 6 years earlier. The physical examination revealed a painless non-ulcerated right palatine tonsillar swelling and a 2×3.5 cm right submaxillary lymph node. Pharyngeal computed tomography (CT) showed an enhanced enlarged right tonsil and right submaxillary lymph node. Preoperatively, a routine X-ray and CT of the chest showed a mass in the lower lobe of the right lung, suggesting tonsillar metastasis from the lung. A right tonsillectomy was performed. The postoperative pathology showed that the lesion contained small tumor cells arranged in irregular nests with infiltrative growth and increased mitotic activity. Immunohistochemically, the tumor was positive for CD56, chromogranin A (CHGA), cytokeratin 18 (CK18), and Ki-67 (90%) (Figure 1). The diagnosis was right-tonsillar SCNC. Subsequently, the patient underwent fine-needle aspiration cytology (FNAC) of the lung mass, which revealed SCNC. PET/CT was performed to detect whether the patient had other distant metastases. 18F-FDG PET/CT images revealed multiple regions of increased FDG activity in the right lung (SUVmax=11.6) and the right oropharynx (SUVmax=7.3). In addition, multiple intensely 18F-FDG-avid right submaxillary (SUVmax=13.6) and mediastinal (SUVmax=7.9) lymph nodes were detected (Figure 2). The FDG levels in other areas were not elevated. A diagnosis of a primary tumor of the lung with widespread metastases was suggested. After two cycles of gemcitabine (1000 mg/m ^2 ^, once per week×3) plus carboplatin (200 mg/m ^2 ^×1), brain metastasis was detected on CT. The patient died of multiple metastases after 6 months. 

## 3. Discussion

Primary extrapulmonary small-cell carcinomas are extremely rare (8). Only two cases involving the palatine tonsils have been reported (9). Most neuroendocrine carcinomas of the palatine tonsils metastasize from other sites, mainly small-cell lung carcinoma (10). The 2004 World Health Organization (WHO) classification recognizes four major types of neuroendocrine tumor (NET) of the lung: typical carcinoid (TC), atypical carcinoid (AC), large-cell neuroendocrine carcinoma (LCNEC), and small-cell lung cancer (SCLC) (11). SCLC is subdivided into highly, intermediately, and poorly differentiated grades (11). Our patient had poorly differentiated SCLC. The prognosis of typical SCLC is still measured in months, and long-term survival is extremely unusual (the 5-year survival is less than 5%) (12). Two-thirds of the patients present with distant metastases, and these individuals have poorer survival rates (13). The mean interval between the development of the primary lung carcinoma and the appearance of tonsillar metastasis is 8 months (7); the mean interval between the appearance of tonsillar metastasis and death is 5 months (14). Our patient was given chemotherapy, but died of multiple distant metastases after 6 months.

The manner in which tonsillar metastases evolve remains unknown. There are four hypothetical pathways for metastases: 1) hematogenous spread via the arterial systemic circulation (6); 2) By-passing the lungs through the paravertebral plexus (6). 3) Since the palatine tonsils do not have afferent lymphatic vessels, only cells transported retrogradely could arrive (2). PET/CT showed high FDG uptake in multiple right submaxillary and mediastinal lymph nodes in this case, suggesting lymphatic spread. 4) The possibility of direct implantation of cancer cells from instrumentation during bronchoscopy has been suggested in patients with lung cancer (14). However, the enlarged right tonsil in our patient was found before bronchoscopy similar to the case reported by Mastronikolis et al. (6).

The diagnostic palatine tonsillar metastasis from the lung is difficult. The metastatic lesion in the palatine tonsil remains undetected by conventional diagnostic imaging, such as computed tomography (CT), magnetic resonance imaging (MRI). As many previous reports, primary sites were usually detected before palatine tonsillar metastases (2, 3, 5-7, 10, 15). In our case, the preoperative pharyngeal CT and chest x-ray showed masses in the right palatine tonsil, right submaxillary lymph node, and right lung. These findings could not rule out lymphoma. A right tonsillectomy was performed and the pathology demonstrated right palatine tonsillar SCNC. Therefore, we suspected metastasis from the lungs. PET/CT supported this hypothesis and we found widespread metastases of the primary lung tumor. Subsequently, the lung FNAC revealed SCNC. These findings suggested that lung surgery was not useful and predicted a poor prognosis. To our knowledge, this is the first reported case of SCLC metastatic to the tonsil after tonsillectomy seen on 18F-FDG PET/CT. The findings avoided unnecessary aggressive treatment. Unfortunately, the patient died 6 months later after two cycles of chemotherapy.

Although tonsillar metastasis is a systemic malignant tumor metastasis, treatment should be considered, because long-term survival may be possible (2). The rather poor outcome of patients with palatine tonsillar metastases is due to the lack of effective treatments. Comprehensive treatment usually combines chemotherapy, radiotherapy, and surgery. Although a few patients have achieved relatively good results (15), most cases present at a very late stage of the disease and have a very small chance of cure with radiotherapy (6). The 2012 National Comprehensive Cancer Network (NCCN) guidelines recommend adjuvant chemotherapy alone for p-N0 SCLC patients who undergo complete resection, while concurrent chemotherapy and mediastinal radiotherapy should be considered in patients with lymph node metastasis (16).

It is very important to stage SCLC accurately. FDG-PET has a valuable role in detecting and characterizing neuroendocrine carcinoma of the lung (17, 18), albeit mainly in non-small-cell lung cancer (19, 20). Few reports have focused on SCLC (17, 18) and those that have done so are inconclusive (19, 20). Orcurto et al. (18) demonstrated a cardiac metastasis in a case of SCLC using FDG PET/CT (18). Oh et al. found that the whole-body metabolic tumor volume (WBMTV) provided useful information for evaluation of the stage and prognosis of SCLC in a series of 106 patients, and ultimately in the selection of the appropriate therapy for each patient group (17).

In conclusion, this case did not have specific findings, but various modalities in their evaluation are important for correct staging of the masses. In our case, PET/CT may have helped accurate staging, guided the management, and predicted the prognosis. To our knowledge, this is the first reported case of SCLC metastatic to the tonsil seen on 18F-FDG PET/CT.
